# Accumulation of copper and lead in ruminants grazing on a contaminated shooting range in Nordland County, Norway

**DOI:** 10.1007/s11356-023-31609-y

**Published:** 2024-01-13

**Authors:** Ida Vaa Johnsen, Jorunn Aaneby

**Affiliations:** Norwegian Defence Research Establishment (FFI), Instituttveien 20, NO-2007 Kjeller, Norway

**Keywords:** Copper, Grazing, Heavy metal, Lead, Ruminants, Shooting range, Soil contamination

## Abstract

Shooting ranges contain copper (Cu) and lead (Pb) contamination, which can be a risk for grazing ruminants. This study examines the accumulation of lead and copper in blood of lambs and calves, as well as in the liver of lambs. It compares these results with those of a previous study, which calculated the ingested dose of copper and lead based on soil ingestion and concentration in soil and plants. Blood samples were collected both before and after the grazing period that lasted from late May to mid-September. Liver samples were obtained during the slaughter of the lambs in the fall. Out of 61 liver samples, only one (3.7 mg Pb/kg dw) exceeding the presumed normal level in lamb liver of 3 mg/kg (dw). Copper concentrations exceeding the normal (300 mg/kg dw) concentration was found in 14 of the liver samples (341–1877 mg Cu/kg dw). Among these, two liver samples (1069 and 1877 mg Cu/kg dw) exceeded the level at which sheep are poisoned (1000 mg/kg dw). There was no statistically significant difference in the copper and lead concentration in liver of lambs that did and did not have the shooting range as part of their pasture. The average concentration of copper (lamb: 1.1 ± 0.37 µg Cu/g, calves: 0.6 ± 0.16 µg Cu/g) and lead (lamb: 0.010 ± 0.008 µg Pb/g calves: 0.01 ± 0.014 µg Pb/g) in the blood samples collected from the lambs and calves did not exceed the upper limit of what is considered normal (sheep: 1.35 mg Cu/kg and 0.3 mg Pb/kg, cattle: 1.7 mg Cu/kg and 0.35 mg Pb/kg). Copper concentration in the blood was notably higher in samples collected from the sheep before (1.3 ± 0.35 µg Cu/g) compared to after (0.8 ± 0.22 µg Cu/g) the grazing period. No statistically significant difference was found in lead and copper concentrations in the blood of lambs and calves grazing inside (lamb: 0.7 ± 0.21 µg Cu/g and 0.01 ± 0.008 µg Pb/g, calves: 0.6 ± 0.16 µg Cu/g and 0.02 ± 0.020 µg Pb/g) and outside (lamb: 0.9 ± 0.21 µg Cu/g and 0.13 ± 0.008 µg Pb/g, calves: 0.6 ± 0.17 µg Cu/g and 0.009 µg Pb/g) the shooting range. Grazing on areas contaminated by shooting activity did not appear to have any major implications for the accumulation of copper and lead in blood of cattle and sheep, as well as in the liver of sheep. The findings from this study indicate that employing site specific risk assessments for ruminants incorporating soil ingestion represents a viable approach.

## Introduction

Ammunition contains metals such as copper (Cu) and lead (Pb) and spent bullets cause contamination on shooting ranges (Randich et al. 2002; Mariussen et al. 2017). Shooting ranges in Norway, and other countries like Switzerland (Tandy et al. 2017), are often unfenced, allowing access to grazing animals. Therefore, sheep resting and grazing on heavily contaminated areas such as bullet traps is a normal sight on Norwegian shooting ranges (Johnsen et al. 2018; Johnsen and Aaneby 2019). Poisoning of ruminants grazing on contaminated areas has been reported on several occasions (Gudmundson 1993; Guitart et al. 2010; Headley et al. 2008; Krametter-Froetscher et al. 2007; Oruc et al. 2009; Guagnini et al. 2018; Liu 2003). Payne and Livesey (2010) found that approximately 25% of all cases of lead poisoning in grazing animals were attributed to geochemical factors, while about 7% were linked to metallic sources, out of a total of 454 cases. For example, VetRecord (2022, 2023) documented several cases of lead poisoning in cattle while on pasture. In one of the cases, the lead concentrations in the soil exceeded 1000 mg/kg. A well-documented case of lead poisoning resulting from grazing on areas contaminated by shooting activity occurred in Switzerland. Several calves succumbed to lead poisoning after grazing on an old shooting range (Braun et al. 1997). The lead concentration in the soil where the calves had grazed was 29 550 mg/kg. Guagnini et al. (2018) also described lead poisoning in 16 out of a herd of 60 cattle that grazed on a pasture previously used for military artillery in Brazil. 

Chronic lead poisoning in ruminants is rather rare (NAS 1980), but acute poisoning of cattle after ingestion of, for instance, leaded paint or old car batteries, is more common (Leary et al. 1970; Wilkinson et al. 2003). Following lead exposure there is an initial increase in lead concentration in the blood, accompanied by a temporary accumulation of lead in the liver and kidneys. Over time, lead will replace calcium in the bones, leading to long-term storage (Zmudski et al. 1983; Payne and Livesey 2010). Symptoms of acute lead poisoning may include blindness, ataxia, cramps, muscle tremors, aggression, anorexia, salivation, stomach pains, constipation and diarrhea (Sharpe and Livesey 2004). According to Rumbeiha et al. (2001) the half-life of lead in the blood of cattle varied widely, ranging from 48 to 2507 days after accidental poisoning. Table 1 summarises some toxic concentrations and doses of copper and lead for ruminants from literature, in addition to normal and poisonous concentrations in liver and blood.

**Table 1:** Acute and toxic doses, limit value in fodder and normal and poisonous concentrations in liver and blood of copper and lead in ruminants from literature

Reference	Sheep	Cattle	Comment
Lead			
Payne and Livesey (2010)	600–800 mg/kg bw	600–800 mg/kg bw	Acute deadly dose
Payne and Livesey (2010)	6 mg/kg bw/day	6 mg/kg bw/day	Chronic effect
Braun et al. (1997)		160 mg/kg bw/day	Deadly for calves within 5–8 days
Liu (2003)	4.4 mg/kg bw/day		Minimal cumulative deadly dose
Wilkinson et al. (2003)		12 mg/kg bw/day	Deadly after 60 days
NAS (1980)	2 mg/kg bw/day		Safety limit value (60–100 mg/day)
Zmudski et al. (1983)		2.7 and 5 mg/kg bw/day	Sign of poisoning after 20 and 5 days respectively in calves
Rupflin and Krebs (2015)	0.7 mg/kg bw/day	1 mg/kg bw/day	Minimal toxic dose in repeated intake of lead acetate (50 mg/kg [dw] in fodder)
2002/32/EC (2002)	5 mg/kg	5 mg/kg	Limit value in fodder with 12% water content in EU
NAS (1980)	0–3 mg/kg (dw)	0–3 mg/kg (dw)	Normal concentration in liver
	10 mg/kg (dw)	10 mg/kg (dw)	Poisonous concentration in liver
Cowan and Blakley (2016)	0.05–0.25 mg/kg	0.05–0.25 mg/kg	Normal concentration in blood
	> 0.35 mg/kg	> 0.35 mg/kg	Poisonous concentration in blood
Copper			
Bradley (1993)		< 1 mg/kg bw/day	Clinical sign of poisoning after 2.5 years
Perrin et al. (1990)		8 mg/kg kv/day	Chronic poisoning
Oruc et al. (2009)	20–100 mg/kg bw		Acute poisoning
	3.5 mg/kg bw/day		Chronic poisoning, long time exposure
Rupflin and Krebs (2015)	17 mg/kg	40 mg/kg	Limit value in fodder in Switzerland
NAS (1980)	< 300 mg/kg (dw)	< 300 mg/kg (dw)	Normal concentration in liver
	< 1000 mg/kg (dw)	2000–3000 mg/kg (bw)	Poisonous concentration in liver
Borobia et al. (2022)	400–1000 mg/kg (dw)		Suggested marginally toxic range for liver concentration
Buck and Sharma (1969)	0.75–1.35 mg/kg		Normal concentration in blood
Bradley (1993)		0.7–1.7 mg/kg	Normal concentration in blood

Sheep are especially vulnerable to copper poisoning as they only have a small copper storing capacity in the liver (Bradley 1993; Gupta 2018). While acute copper poisoning in sheep can occur, it is rare. Chronic copper poisoning, on the other hand, is more commonly observed in older animals that have accumulated copper in their liver over time (Sivertsen and Plassen 2004; Froslie et al. 1985). Copper poisoning in cattle is rare, but acute cases have been reported, particularly in calves (Bradley 1993; Minervino et al. 2009). According to Otter et al. (2023), cattle may be more susceptible to copper poisoning if they have underlying hepatic damage. Copper poisoning can be divided into two phases: an accumulation phase, during which copper is stored in the liver, and a phase of acute illness. Once the liver's storage capacity is reached, rapid hepatic cell death occurs, releasing copper into the bloodstream (Minervino et al. 2009; Bradley 1993; Borobia et al. 2022). This leads to extensive hemolysis, followed by liver- and kidney failure, which can cause icterus, anemia and asphyxia. Symptoms may include anorexia, runny nose, stomach pain and fatigue (Roubies et al. 2008; Bradley 1993; Minervino et al. 2009). Apart from the copper concentration ingested by ruminants, a deficiency of molybdenum (Mo) in fodder can also lead to copper poisoning. An optimal Cu/Mo ratio is considered to be 6, while a ratio greater than 10 can lead to copper poisoning (Hidiroglou et al. 1984; Buck and Sharma 1969).

The amount of heavy metals ingested by ruminants grazing on contaminated areas depends largely on the grazing behavior of the ruminants, metal accumulation in the grass, soil contamination levels, and the rate of soil ingestion. These data were acquired in a prior study, and used to calculate a theoretical dose (copper and lead) ingested by the ruminants grazing on an area in Norway containing a shooting range (Melbu) (Johnsen and Aaneby 2019). The current study builds upon that research, allowing for a comparison between the actual measured concentrations accumulated in the ruminants and the calculated ingested dose. This was accomplished by collecting blood and liver samples from animals (sheep and cattle) that grazed in Melbu and then analysing the concentration of copper and lead in these samples.

This study aims to compare the copper and lead concentrations in the blood and liver of ruminants (sheep and cattle) with the calculated ingested doses of these metals The results will provide valuable insights for risk assessments concerning ruminants grazing in areas contaminated by lead and/or copper, including locations like shooting ranges, vicinity of metal smelters, or heavily trafficked roads. The hypothesis posits that the levels of copper and lead found in the blood and liver of the ruminants will align with the theoretical ingested doses calculated by Johnsen and Aaneby (2019) for the same animals, in the same area (Table 2). The doses were calculated using the soil ingestion rate, as well as the concentrations of copper and lead found in the soil and grass during the study. For acute doses, it was assumed that the ruminants grazed on the most contaminated areas for 24 h. For chronic doses, it was assumed that the ruminants grazed in the most contaminated areas for 10% of their time (Johnsen and Aaneby 2019). For this case, the theoretical ingested doses were low, and none exceeded the limit for chronic or acute copper or lead poisoning in cattle or sheep (Table 2), therefore it is expected that the concentration of lead and copper in liver and blood is not elevated.

**Table 2:** Calculated chronic and acute ingested doses of lead and copper based on concentration in soil, grass, soil ingestion rate and grazing behaviour from Johnsen and Aaneby (2019)

mg/kg, bw, day		Lead	Copper
		Calculated dose	Calculated dose
Sheep	Chronic	0.12	0.07
	Acute	1.2	0.7
Cattle	Chronic	0.12	0.08
	Acute	1.2	0.8

## Materials and methods

### Area

The study area was an island called Melbu in Hadsel municipal, Nordland County in Norway, this island contains a shooting range. The shooting range area covers approximately 5 km^2^ and includes 10 individual ranges, with 10% of the area affected by shooting activity. The Norwegian Armed Forces (Home Guard) have used the range from the 1950’s until 2005. Some parts of the range are still utilized by civilians. A parallel study investigated soil ingestion rate and heavy metal dose ingested by ruminants (Johnsen and Aaneby 2019). In this study, soil and grass samples were analysed in the shooting ranges and in a reference area outside the range. The soil in the shooting ranges contained up to 1654 ± 556 mg Cu/kg and 3700 ± 684 mg Pb/kg, and the grass contained up to 35 ± 7.5 mg Cu/kg and 52 ± 5.1 mg Pb/kg (Johnsen and Aaneby 2019). In the grass sample with copper concentration of 35 mg/kg, the Cu/Mo-ratio was 7. Elevated Cu/Mo ratios (> 10) were found in several samples, with the highest ratio recorded at 37. Lead concentrations in the soil of the most contaminated areas were significantly lower than the levels that caused acute poisoning in six calves in Switzerland (29,550 mg/kg) (Braun et al. 1997), but exceeded the upper safe limit recommended by the Norwegian Veterinary Institute for lead in soil where ruminants graze (300 mg/kg) (Bernhoft 2011). The copper concentration in the soil in the most contaminated areas were above the safe limit for sensitive land use in the soil quality guidelines set by the Norwegian Pollution Control Authority (100 mg/kg) (now the Norwegian Environment Agency) (Vik et al. 1999). Areas with high shooting activity generally exhibited elevated copper and lead concentrations (> 800 mg/kg), markedly surpassing levels found in the reference area (< 30 mg/kg). Grass with a lead concentration of 2–5 mg Pb/kg dry weight (dw) is considered normal (Chaney 1989; Robinson et al. 2008). The EU’s limit for lead in green fodder is 30 mg/kg (12% water content, 33.6 mg/kg dw) (2002/32/EC 2002). The considered normal level and the EU limit of lead in green fodder was exceeded in the most contaminated areas. A concentration of 3–20 mg Cu/kg (dw) is considered normal in grass (Chaney 1989; Robinson et al. 2008), there is no EU limit for copper in fodder, but Switzerland has a limit of 17 mg/kg (dw) in sheep fodder (Table 1) (Rupflin and Krebs 2015). According to Gupta (2018), copper toxicity has been observed in sheep at concentrations as low as 10 mg/kg. The considered normal copper concentration in grass, and the Swiss limit for copper in sheep fodder was exceeded in the most contaminated areas.

### Sampling

A local veterinarian collected blood samples from both sheep and cattle before and after the grazing season. The grazing season lasted from late May to mid-September. Blood was collected in containers suitable for metal analysis, and with lithium heparin as an anti-coagulant. Blood samples were obtained from 25 lambs intended for grazing on the shooting range and 25 lambs designated for grazing outside the range, both before and after the grazing period. Because of loss, fewer samples were collected after the grazing period than before. Similar samples were gathered from calves, except those expected to graze outside the shooting range prior to the grazing period (due to a misunderstanding between the farmer and the collecting veterinarian). Liver samples from lambs were collected during fall slaughter, totalling 61 livers. Unfortunately, liver samples from calves were not collected because they were not slaughtered until spring. By that time, much of the potential copper and lead accumulation would likely have been excreted. The local farmer was familiar with the usual grazing areas of each animal, and therefore determined from which lambs to collect blood and liver samples. Additionally, some of the sheep were equipped with GPS trackers, providing confirmation of their grazing areas.

### Analysis

The liver samples were frozen directly after slaughter and shipped frozen to the laboratory (at the Norwegian Defence Research Establishment [FFI]). Upon arrival, they were thawed, weighed, and processed. To remove any potential contaminations from the slaughter process, the liver samples were rinsed in purified water (Milli Q). Subsequently, the samples were dried at 60˚C until a stable weight was achieved (approximately 72 h) and then crushed in a ball mill (Retsch RM100) at 500 rpm for 10 min. The digestion method was similar to the method described by Nóbrega et al. (2012); approximately 0.3 g of dried and crushed liver were weighed into Teflon vials, added 7 ml HNO_3_ (Suprapure 67%, Merck) and 0.5 ml H_2_O_2_ (Suprapure 30%, Merck) and digested in a pressurized microwave (Ultrawave [Milestone]) at 220˚C for 10 min. The samples were prepared in triplicates. With every 12 samples, two blanks and one certified reference material for bovine liver (BCR®-185R, European Commission, Institute for Reference Materials and Measurements [As, Cd, Cu, Mn, Pb, Se, Zn]) was digested.

The blood samples were digested using the method described by Harrington et al. (2014). Following vortexing of the blood samples, 250 µl blood (triplicates) was pipetted into Teflon vials, added 2 ml HNO_3_ (Suprapure 67%, Merck) and left under a fume hood for 30 min. The samples were then added 1.5 ml H_2_O_2_ (Suprapure 30%, Merck) and digested in an Ultrawave (Milestone) at 220˚C for 10 min. With every 12 samples, two blanks and one certified reference material for bovine blood (ERM®-CE196, European Commission, Institute for Reference Materials and Measurements [Pb, Cd]) was digested.

All the samples, blanks and reference materials were diluted and analysed for metals using an ICP-MS (inductively coupled mass spectrometer) (Thermo X-series II). A four-point standard curve and an internal standard was used for determination of the metal concentration in the samples. For further quality assurance, three certified reference materials for water were used (TMDA-53.3, TM-23.4 [Al, Sb, Cd, Cr, Co, Cu, Fe, Pb, Mn, Ti, Zn] and AES-07 [Al, Ca, Mg, K, Na], from Environmental Canada).

### Statistics

In all cases where statistical analysis was performed, the Shapiro–Wilk test of normality was done (SPSS). A parametric test was performed in normally distributed datasets, a non-parametric test was performed in non-normally distributed datasets and both parametric and non-parametric test were performed in datasets that had varying degree of normal distribution. 

### Health risk assessment 

To assess the health risk of the ruminants, the concentration of copper and lead in the blood and liver samples were compared to known values from literature associated with poisoning (Table 1). To assess the health risk associated with human consumption, the concentration of lead in the lamb liver was compared to the limit value for lead in offal set by the EU (EC-Regulation-1881/2006 2006). A short literature review was also performed to find the correlation between lead in liver and meat, since meat is a more important pathway for human exposure than liver.

## Results and discussion

### Liver

Out of the 61 liver samples collected from the lambs, the grazing location was unknown for 28 lambs, 19 had grazed inside the shooting range, and 14 had not grazed on the shooting range. Table 3 displays the concentrations of copper and lead in the livers.

**Table 3:** Concentration (dw) of copper and lead measured in lamb liver collected from lambs that had grazed in Melbu. Some of the lambs had the shooting range as part of their pasture, some lambs did not graze on the shooting range, and it was unknown where the last group of lambs had grazed. Concentrations exceeding the normal concentrations (Table 1) are marked in bold

	On range		Outside range		Unknown	
	Cu	Pb	Cu	Pb	Cu	Pb
	mg/kg	mg/kg	mg/kg	mg/kg	mg/kg	mg/kg
	**357**	1.4	34	1.7	**710**	0.9
	88	0.8	62	2.5	133	0.8
	54	1.3	49	1.6	**481**	2.4
	224	1.1	110	0.4	55	1.8
	**381**	0.8	**554**	0.6	149	1.8
	284	1.2	**459**	0.3	72	2.0
	149	1.0	207	0.4	**1069**	2.2
	**1877**	2.0	132	0.4	239	1.4
	67	2.5	**396**	1.6	132	0.8
	96	1.3	**376**	0.4	70	2.9
	55	0.6	152	0.3	2	1.4
	72	0.8	203	0.2	1	1.5
	290	0.6	**517**	0.7	**432**	1.6
	84	**3.7**	143	0.7	113	1.3
	81	0.6			216	1.2
	113	0.6			191	0.8
	160	0.9			**418**	1.6
	110	0.5			65	1.6
	115	0.2			98	0.9
					**341**	0.7
					97	0.9
					101	2.1
					124	0.3
					105	0.4
					99	0.1
					100	0.9
					203	1.1
					282	0.7
Average	245	1.2	242	0.8	218	1.3
SD	408	0.81	181	0.72	232	0.66

The average residual weight of the liver samples post-desiccation was 32% of their initial mass. Only one liver sample had a lead concentration (3.7 mg Pb/kg [dw], equivalent to 1.2 mg/kg in wet weight [ww]) exceeding the presumed normal lead concentration in liver of lamb of 3 mg Pb/kg (dw) (NAS 1980). This liver was collected from a lamb that was likely to have the shooting range as part of its pasture. Although the concentration exceeded the 'normal' lead concentration in sheep liver, it was significantly lower than the concentration at which poisoning occurs (10 mg Pb/kg [dw]) (NAS 1980). These findings suggest that the sheep’s intake of lead from the shooting range is not compatible with poisoning or accumulation. In comparison, the lead concentration in the liver of lead poisoned cattle in Scotland, described in VetRecord (2022) was 51.1–109 mg/kg (ww). 

Copper levels above the normal range (> 300 mg/kg [dw]) (NAS 1980) were observed in 14 of the liver samples (ranging from 341 to 1877 mg Cu/kg [dw]). In two liver samples (1069 and 1877 mg Cu/kg [dw]), the copper concentration surpassed the threshold indicating potential poisoning in sheep (1000 mg/kg [dw]) (NAS 1980), as well as the range where copper may be released from the liver into the bloodstream (1000–3000 mg/kg)(Gupta 2018). One of the lambs with poisonous concentration of copper in the liver had grazed on the shooting range, while it was uncertain where the other lamb had grazed. There was no statistically significant difference (p > 0.5) in the metal concentrations (lead and copper) in the livers of lambs that grazed solely outside the shooting range, and those that included the shooting range in their pasture. This was the case for both the parametric (ANOVA, Excel) and non-parametric tests (Mann–Whitney, SPSS). The results suggest that elevated copper concentrations in the livers of some lambs were likely unrelated to their grazing location. In a study involving 599 lambs grazing in six different areas across Norway, the average hepatic copper concentration varied from 5 to 240 mg/kg (ww) (corresponding to 15–750 mg/kg [dw], with 32% dry weight) (Sivertsen and Plassen 2004). This was in sheep herds, with no expected copper poisoning. In Sivertsen and Plassen (2004), one sheep herd grazed in an area close to Melbu (Sortland, in Vesterålen municipal, Nordland county), the mean hepatic copper concentration in this herd was 57 (5–230) mg/kg (ww) (corresponding to 178 (15–720) mg/kg [dw], with 32% dry weight). These results further strengthen the assumption that the hepatic copper concentrations found in sheep grazing on Melbu (245 mg/kg on range, 242 mg/kg outside range and 218 mg/kg unknown grazing area [dw]), was independent of the grazing area.

### Blood

The average concentration of copper (lamb: 1.1 ± 0.37 mg Cu/kg, calves: 0.6 ± 0.16 mg Cu/kg) and lead (lamb: 0.010 ± 0.008 mg Pb/kg calves: 0.01 ± 0.014 mg Pb/kg) in the blood samples collected from both lambs and calves (Table 4 and 5) did not exceed the upper limit considered normal (sheep: 1.35 mg Cu/kg and 0.3 mg Pb/kg, cattle: 1.7 mg Cu/kg and 0.35 mg Pb/kg) (Buck and Sharma 1969; Bradley 1993; Cowan and Blakley 2016; NAS 1980). Elevated copper concentrations were observed in 13 blood samples from lambs (ranging from 1.4 to 2.4 mg Cu/kg); notably, all elevated values were from samples collected prior to the grazing period. None of the lambs or calves had elevated concentrations of lead in the blood.

**Table 4:** Concentration of copper and lead in blood from lambs both before and after the grazing period. One group of lambs had the shooting range as part of their pasture, and the other group did not graze on the shooting range. Concentrations exceeding the normal concentrations (Table 1) are marked in bold

Lamb no	On range				Lamb no	Outside range			
	Before grazing		After grazing			Before grazing		After grazing	
	Cu	Pb	Cu	Pb		Cu	Pb	Cu	Pb
	mg/kg	mg/kg	mg/kg	mg/kg		mg/kg	mg/kg	mg/kg	mg/kg
1	1.3	0.01	0.67	0.01	28	1.1	0.01	0.38	0.01
2	1.1	0.01	0.66	0.02	29	0.9	0.03	0.73	0.02
3	1.1	0.02			30	1.2	0.01	0.95	0.02
4	1.1	0.01	0.67	0.01	31	**2.1**	0.02	0.82	0.02
5	0.99	0.01			32	1.1	0.02		
6	0.92	0.00			33	**2.1**	0.03		
7	1.1	0.01	0.69	0.02	34	**2.4**	0.01		
8	0.99	0.01	0.80	0.00	35	0.9	0.01		
9	1.3	0.02	0.77	0.02	36	1.2	0.00	0.83	0.02
10	**1.4**	0.01	0.87	0.01	37	**1.7**	0.02	0.84	0.03
11	0.95	0.00			38	0.9	0.00	1.04	0.00
12	1.3	0.00	0.72	0.02	39	1.3	0.01	0.84	0.01
13	1.3	0.00	0.99	0.00	40	1.2	0.01		
14	0.9	0.01			41	1.3	0.00	0.81	0.01
15	**1.4**	0.01	0.77	0.02	42	1.0	0.00		
16	**1.4**	0.03	0.35	0.00	43	0.9	0.01		
17	1.2	0.02	0.58	0.01	44			0.90	0.01
18	**1.5**	0.00	0.86	0.01	45	1.1	0.01	1.11	0.02
19	0.98	0.01	1.14	0.02	46	1.2	0.00	0.62	0.01
20	1.2	0.01	0.45	0.00	47	1.1	0.00	0.99	0.01
21	**2.0**	0.01	1.15	0.01	48	**1.6**	0.00	0.90	0.01
22	1.0	0.02			49	**1.4**	0.00		
23	**1.8**	0.01			50	1.0	0.00		
24	1.0	0.00	0.61	0.01	51			1.22	0.00
25			0.57	0.01	52	1.3	0.00		
26	**1.6**	0.01			53	0.8	0.00	1.25	0.01
27			0.58	0.01	54	0.9	0.00	0.86	0.02
Average	1.2	0.011	0.7	0.01		1.3	0.011	0.9	0.013
SD	0.27	0.007	0.21	0.008		0.41	0.008	0.21	0.008

**Table 5:** Concentration of copper and lead in blood from calves both before and after the grazing period. One group of calves had the shooting range as part of their pasture, and the other group did not graze on the shooting range

Calves no	On range				Calves no	Outside range	
	Before grazing		After grazing			After grazing	
	Cu	Pb	Cu	Pb		Cu	Pb
	mg/kg	mg/kg	mg/kg	mg/kg		mg/kg	mg/kg
1	0.7	0.02	0.51	0.02	26	0.41	0.01
2	0.5	0.05	0.50	0.01	27	0.55	0.01
3	0.4	0.00	0.44	0.01	28	0.85	0.01
4	0.8	0.01	0.48	0.01	29	0.46	0.00
5	0.5	0.02	0.69	0.00	30	0.52	0.00
6	0.6	0.00	0.53	0.00	31	0.46	0.00
7	0.5	0.00	0.45	0.00	32	0.33	0.00
8	0.3	0.01	0.43	0.01	33	0.78	0.00
9	0.6	0.00	0.65	0.02	34	0.40	0.01
10	0.6	0.01	1.01	0.10	35	0.51	0.00
11	0.5	0.00	0.54	0.03	36	0.69	0.01
12	0.5	0.00	0.46	0.03	37	0.34	0.00
13	0.6	0.00	0.53	0.02	38	0.69	0.02
14	0.6	0.00	0.88	0.04	39	0.61	0.02
15	0.6	0.01	0.76	0.03	40	0.51	0.03
16	0.4	0.01	0.71	0.01	41	0.59	0.01
17	0.6	0.01	0.37	0.01	42	0.90	0.02
18	0.5	0.00	0.36	0.02	43	0.77	0.02
19	0.5	0.01	0.77	0.02	44	0.74	0.03
20	0.5	0.00	0.64	0.01	45	0.62	0.01
21	0.4	0.01	0.55	0.02	46	0.46	0.01
22	0.9	0.01	-	-	47	0.33	0.00
23	0.6	0.01	0.58	0.02	48	0.54	0.01
24	0.4	0.01	0.58	0.02	49	0.77	0.01
25	0.9	0.00	0.66	0.00	50	0.85	0.00
Average	0.6	0.01	0.6	0.02		0.6	0.009
SD	0.14	0.011	0.16	0.020		0.17	0.0093

The copper concentration was significantly higher (p < 0.05, paired t-test [Excel] and Wilcoxon signed rank test [SPSS]) in the blood collected from the sheep before the grazing period (average 1.3 ± 0.35 mg Cu/kg) compared to the blood collected after the grazing period (0.8 ± 0.22 mg Cu/kg). This suggests that the sheep may be ingesting copper while at the farm, either from the fodder or another source. Alternatively, it is possible that the fodder at the farm was deficient in molybdenum. Molybdenum is an important factor in copper poisoning, as it binds copper through the digestion system, making it unavailable for uptake (Hidiroglou et al. 1984; Buck and Sharma 1969). If fodder contains insufficient molybdenum compared to copper (Cu/Mo > 10), copper poisoning might occur. If the fodder contains too much molybdenum compared to copper (Cu/Mo < 6), copper deficiency might occur (Villar et al. 2002). No statistically significant difference was observed in the copper concentrations in the blood from calves collected before and after the grazing season. This held true for both statistical tests performed.

No statistically significant difference (p > 0.05) was observed in the lead concentration between sheep and cattle before (lambs: 0.009 ± 0.0086 mg Pb/kg, calves: 0.01 ± 0.011 mg Pb/kg) and after (lambs: 0.12 ± 0.0076 mg Pb/kg, calves: 0.02 ± 0.02 mg Pb/kg) the grazing period when analysed using the paired t-test (Excel). However, such a difference was detected with the Wilcoxon signed rank test (SPSS). The lead concentration was highest in both sheep and cattle after the grazing period. This suggests that the animals ingested lead during grazing in Hadsel. However, it cannot be definitively concluded that the lead originated solely from the shooting range. Animals commonly ingest lead from various sources during grazing, including used car batteries and old leaded paint (Leary et al. 1970; Payne and Livesey 2010). Cowan and Blakley (2016) compiled data on lead testing in tissue and blood of cattle following suspected lead poisoning cases in Canada from 1998 to 2013. A total of 1382 blood samples were recorded. In animals suffering from lead poisoning, the average (n = 301) concentration in blood was 1.33 (0.32–17.9) mg/kg. Blood samples The lead concentrations found in the blood of calves in Melbu fell within the range (0–0.34 mg/kg) observed in cattle not susceptible to lead poisoning according to Cowan and Blakley (2016).

No statistically significant differences were observed in the lead and copper concentrations in the blood of lambs and calves between those grazing inside (lamb: 0.7 ± 0.21 mg Cu/kg and 0.01 ± 0.008 mg Pb/kg, calves: 0.6 ± 0.16 mg Cu/kg and 0.02 ± 0.020 mg Pb/kg) and outside (lamb: 0.9 ± 0.21 mg Cu/kg and 0.13 ± 0.008 mg Pb/kg, calves: 0.6 ± 0.17 mg Cu/kg and 0.009 mg Pb/kg) the shooting range when analysed using ANOVA (Excel). When applying the Mann–Whitney test (SPSS), a significant difference was observed only for lead concentration in the blood of calves between those grazing inside and outside the shooting range. This suggests a potential influence of grazing location on lead exposure. The highest concentration of lead was found in the blood of calves that had the shooting range as part of their pasture. None of the calves had a lead concentration in the blood consistent with poisoning, or exceeding what is considered normal. The findings suggest that calves may ingest trace amounts of lead while grazing on the shooting range. However, in this instance, the dose was minimal and is unlikely to have any discernible impact on the animals' health. In comparison Pareja-Carrera et al. (2014) found significantly (five times) higher lead concentrations in sheep that had grazed on an area contaminated by mining pollution, compared to sheep that had grazed on a reference area.

### Concentration of copper and lead in liver and blood compared to ingested doses of the metals

Johnsen and Aaneby (2019) observed low ingested doses (Table 2), and none exceeded the threshold for chronic or acute copper or lead poisoning in either cattle or sheep (Table 1) (Oruc et al. 2009; Payne and Livesey 2010; Perrin et al. 1990; NAS 1980). This aligns with the findings of this study, where the lead concentrations in both the blood and liver of sheep and cattle did not surpass levels indicative of poisoning. Even though the shooting range was heavily contaminated with lead, up to an average of 3700 mg/kg in larger areas (300 m^2^)(Johnsen and Aaneby 2019), all the lead concentrations in blood and liver, except the concentration in the liver of one sheep, was within what is considered normal. This suggests that soil heavily contaminated with lead may not pose a significant risk to ruminants. However, it is crucial to acknowledge that this study pertains to a specific area, and while the findings hold true for this location, they may not be universally applicable to all areas. Contaminated shooting ranges can exhibit high concentrations of lead and copper. However, these areas with elevated concentrations are typically limited in size. As a result, the risk to ruminants may be higher in locations where contamination is distributed over a larger area. In a study conducted by Pareja-Carrera et al. (2014), lead concentration in the livers of sheep that had grazed on an area contaminated by mining ranged from 0.26–59.74 mg/kg (dw). This demonstrates that sheep can accumulate lead in their livers when exposed to extensive areas with high concentrations. Liu (2003) observed that sheep grazing on an area polluted by a metal smelter in China ingested lead at rates ranging from 1.7 to 21.4 mg Pb/kg bw/day. This led to affliction in 25–40% of the animals, with a mortality rate of 65% among the affected. The calculated dose exceeded that computed in Johnsen and Aaneby (2019) (Table 2). Notably, the lead concentration in the soil (312 mg Pb/kg [dw]) was markedly lower than in Melbu (maximum 3700 mg Pb/kg [dw]). However, it's worth noting that the maximum lead concentration in forages reported by Liu (2003) (180 mg Pb/kg [dw]), greatly surpassed that in Melbu (52 mg Pb/kg [dw]). Liu (2003) found up to 15.3 mg Pb/kg (dw) in liver samples, while the maximum lead concentration found in liver from sheep grazing in Melbu was 3.7 mg Pb/kg (dw). In this study, the lead and copper concentration refers to the total (acid-digested) concentration in both soil and grass. The uptake of heavy metals by ingestion is dependent on the bioavailability of the metal and might vary between areas according to the availability (Peijnenburg and Jager 2003). Furthermore, the bioavailability of copper and lead in soil will impact their uptake in grass, subsequently influencing the dose ingested by ruminants. 

Regarding copper, it's worth noting that many blood samples from sheep exhibited concentrations above the normal range, albeit this occurred before the grazing period. This implies that the sheep did not ingest elevated or toxic doses of copper during the grazing period, but rather ingested elevated doses from a source on the farm. Instances of copper poisoning from farm fodder have been documented. For instance, Perrin et al. (1990) reported a case where 44 cattle succumbed after consuming fodder with copper concentrations ranging from 400–500 mg Cu/kg (dw) due to a production error. Bradley (1993) identified cases of copper poisoning in cattle following the consumption of fodder with copper concentrations of up to 37.5 mg Cu/kg (dw). Notably, this level is below the Swiss guideline values for copper in cattle feed, which stand at 40 mg/kg (dw) (Table 1). Nine animals in a herd of 63 died over a two-year period. A similar case for sheep were investigated by Hidiroglou et al. (1984). The copper concentration in the liver were as high as 500 mg/kg in some sheep showing signs of copper poisoning. The fodder concentration was only 9.8–13.6 mg Cu/kg (dw), which is lower than the Swiss guidline for copper in sheep fodder (17 mg Cu/kg [dw]) (Table 1). Similarly, in Melbu, there were sheep exhibiting elevated concentrations of copper in the liver. It's important to note that the liver accumulates copper gradually over time, and it's plausible that the source of this copper could originate from either the farm or the shooting range. This coincides well with the findings from Johnsen and Aaneby (2019), where it was found that the copper intake in ruminants is quite (but not completely) independent of the copper concentration in the soil for two reasons: low soil ingestion rate, and no correlation between the copper concentration in soil and grass. The maximum copper concentration found in the grass in Melbu (35 mg/kg [dw]) was higher than the copper concentration (9.8–13.6 mg/kg [dw]) that caused poisoning in the sheep investigated by Hidiroglou et al. (1984). The Cu/Mo-ratio in the fodder was very high (32 to 136) which contributed to the copper poisoning, while the Cu/Mo-ratio in Melbu was between 4–37. Even though the Cu/Mo-ratio in Melbu was high in some of the samples, this is over a smaller area. Smith et al. (2009) monitored sheep grazing on a contaminated floodplain pasture in mid-Wales. The median metal concentration in sheep blood was 1049 µg Cu/L and 46 µg Pb/L (equivalent to ca. 1 mg Cu/kg and 0.046 mg/kg if we assume 1 L blood = 1 kg), which higher than what was found in sheep blood in Melbu (0.7 mg Cu/kg and 0.01 mg Pb/kg) after grazing on the contaminated range. The copper concentration in the soil (maximum of 76 mg Cu/kg [dw]) and plants (maximum of 11 mg Cu/kg [dw]) in mid-Wales was however much lower than what was found in Melbu (maximum of 1654 [soil] and 35 [plant] mg Cu/kg [dw]). The lead concentration in soil and plants in mid-Wales (maximum 75 [plant] and 2940 [soil] mg Pb/kg) and Melbu (maximum 52 [plants] and 3700 [soil]) was quite similar. The reason why lower concentrations of copper and lead was found in blood from sheep grazing in Melbu than in mid-Wales despite the soil and plant concentrations in Melbu being similar or higher than in mid-Wales, can be that the ingested dose was smaller due to lower soil ingestion rate in Melbu. Smaller contaminated areas can also contribute to these results. Smith et al. (2010) found soil ingestion rates ranging from 8.3–20.1%, while Johnsen and Aaneby (2019) found soil ingestion rates < 2%. The comparison of the results from these studies indicate that employing site specific soil ingestion rate for doses calculations provides a reliable indication of the risk associated with metal poisoning (Johnsen and Aaneby 2019; Smith et al. 2009; Smith et al. 2010).

### Implications for human consumption

The limit value for lead in offal for human consumption is 0.5 mg/kg (ww), equivalent to approximately 1.6 mg/kg (dw) (32% [dw]) (EC-Regulation-1881/2006, 2006), while the normal value for lead in sheep liver is < 3 mg/kg (dw) (NAS 1980). This implies that although the lead concentration may fall within what is considered normal, it can still surpass the established limit for human consumption. In this study, the lead concentration in sheep livers exceeded 1.6 mg/kg (dw) in 15 out of 61 samples, for sheep that had grazed both within and outside the shooting range area. It's worth noting that the lead concentration in lamb livers may surpass the defined limit for human consumption, even if the animals haven't been subjected to particularly elevated lead concentrations. In this study, no muscle samples were collected. However, several studies have collected samples from both liver and muscle and looked at the lead concentration. Studies performed by MacLachlan et al. (2016); Pareja-Carrera et al. (2014) and Falandysz (1991) found that the lead concentration in sheep liver was between 3.7–7.5 times higher than in muscle, all the sheep in these studies had been grazing on uncontaminated areas. Pareja-Carrera et al. (2014) also measured lead concentration in sheep that had grazed on an area contaminated by mining. In this area, the concentration in muscle was not affected much (0.04 mg/kg in reference area and 0.08 mg/kg in mining area), but the lead concentration in the liver was 77 times higher than the concentration in the muscle tissue. This suggests that muscle (meat) from sheep (as well as other grazing animals) is safe for consumption, even if they have been grazing on heavily contaminated shooting ranges or other lead-contaminated areas. Small amounts of offal (liver in this case) should also not be a risk, as the concentrations in this case only just exceeded the EU limit for lead in offal for human consumption. However, consumers should be cautious and not consume large amounts of offal from grazing animals, especially vulnerable individuals such as children and pregnant women should avoid consumption. 

There is no limit value for copper in food for human consumption because copper has low toxicity to humans. 

## Conclusion 

Grazing on areas contaminated by shooting activity did not appear to have significant implications for the accumulation of copper and lead in the blood of cattle and sheep, as well as in the liver of sheep. This suggests that ruminants grazing on the Melbu shooting range face minimal risk. The study also underscores the importance of farmers monitoring the concentrations of copper and molybdenum in fodder.

The concentrations of lead in some of the liver samples exceeded the limit value in offal for human consumption in EU. Particularly vulnerable individuals, such as children and pregnant women, should thus restrict their consumption of offal from grazing animals. Given that muscle accumulates considerably less lead than liver, this study asserts that meat from grazing animals, even those on a contaminated shooting range, is safe for human consumption.

The findings from this study indicate that employing site specific risk assessments for ruminants incorporating soil ingestion, and soil and plant concentration of metals to calculate ingested dose, represents a viable approach. To further enhance the risk assessment, bioavailability of the metals in the soil and plants could be implemented into the method.

## Data Availability

Data is available upon request.
